# Study of Plasmid-Mediated Extended-Spectrum Beta-Lactamase-Producing Clinical Strains of Enterobacteriaceae From Tabuk Region

**DOI:** 10.7759/cureus.40183

**Published:** 2023-06-09

**Authors:** Turki Mushabab T Alqahtani, Abdulrahman A Alelyani, Maisaa Mokhtar M Yousuf, Wejdan Mohammed K Alhujayri, Fohad M Husain, Mohammad Zubair

**Affiliations:** 1 Faculty of Medicine, University of Tabuk, Tabuk, SAU; 2 Food Science and Nutrition, King Saud University, Riyadh, SAU; 3 Medical Microbiology, University of Tabuk, Tabuk, SAU

**Keywords:** enterobacteriaceae, e. coli, gram-negative microbes, extended spectrum beta-lactamase (esbl), prevalence study

## Abstract

Background: Extended-spectrum beta-lactamase (ESBL)-producing Enterobacteriaceae threaten infection treatment globally. This study aims to assess ESBLs-E prevalence and multidrug-resistant organisms (MDR) in clinical specimens from Tabuk, KSA.

Methods: A cross-sectional research was carried out in March-May 2023. A collective of 90 Enterobacteriaceae isolates were identified from clinical specimens. The specimen was identified by standard methods. The Enterobacteriaceae member was screed for ESBL production by screening and confirmatory as per the Clinical and Laboratory Standards Institute (CLSI).

Result: *E. coli *was the most common isolate, followed by *Proteus mirabilis and Citrobacter sp*, *Klebsiella oxytoca*, *Klebsiella pneumonia*, *Proteus vulgaris* and *Morganella morganii*. Among the sample, the majority of isolates were from urine (47.8%) followed by pus (25.6%) and the least from other body fluids (6.7%). The *E. coli* showed the highest average antibiotic resistance (73.7%) among all the antibiotics used followed by *P. mirabilis* (70.4%), *K. pneumoniae* (70%), *P. vulgaris* (69.8%), *M. morganii* and *Citrobacter* (69.4% both), and *K. oxytoca* (68.8%). There was a 41.2% average reduction in ESBL positivity from phenotypic to confirmatory test results. The highest reduction was observed among *M. morganii* (66.7%) and the least was observed in *E. coli* (17.1%).

Conclusion: Most of the ESBL-producing isolates were found mainly in blood and urine samples. The most frequent ESBL-producing Enterobacteriaceae were *K. pneumoniae* and *E. coli*. The best options for treating Enterobacteriaceae that produce ESBL are Amoxicillin, Amikacin, and Cefoxitin. ESBL-producing isotopes showed a high resistance rate to cefepime and cefotaxime compared to non-ESBL producers. It is of utmost importance to implement reliable infection control measures in healthcare institutions nationwide.

## Introduction

Enterobacteriaceae members are most common in nosocomial infections. Because Enterobacteriaceae are often highly drug-resistant, the treatment of these bacterial infections can be challenging. There are several diseases which can be caused by them, ranging from urinary tract infections to sepsis. The most common antibiotics used for Enterobacteriaceae were third-generation cephalosporin, quinolones and aminoglycosides. Due to the extensive use of beta-lactam antibiotics, the number of Enterobacteriaceae that are resistant to these antibiotics has increased considerably. Beta-lactamases (especially extended-spectrum beta-lactamases (ESBL)) are the major mechanism of resistance to beta-lactam antibiotics, and they inactivate beta-lactam antibiotics. This is the main reason Enterobacteriaceae are resistant to beta-lactam antibiotics. Enterobacteriaceae, which produce ESBL, are important members of antibiotic-resistant bacteria that cause hospital infections and infections acquired by the community [1].

ESBLs are enzyme produced by bacteria which is also inhibited by beta-lactamase inhibitors such as clavulanic acid. A new trend is reported which is high resistance by Enterobacteriaceae [2-4]. ESBLs are mainly found in the genus *Klebsiella *and *E. coli*, also other genera such as *Enterobacteria, Proteus, Citrobacter, Morganella, Providencia, Salmonella, *and *Serratia* also [5].

ESBLs are plasmid mediated, very easy jump thein genes among Enterobacteriaceae members. This phenomenon of genes is not restricted to beta-lactams, but also to other antibiotics commonly, like fluoroquinolones, aminoglycosides, and sulphonamides [6,7], and because of the above phenomenon, patients required carbapenem antibiotic treatment [2,8]. The extensive use of carbapenem and lead carbapenem-resistant in Enterobacteriaceae [9]. There was a limited antibiotic regimen (e.g. carbapenem, colistin, and Tigecycline) for the ESBL-producing bacterial infections but their in vitro efficacy and toxicity are still unknown [10]. It is necessary to assess the local scenario of ESBL-producing Enterobacteriaceae to understand the burden of the disease and the epidemiology, and to develop and periodic review of hospital infection control strategies to prevent the spread of these bacteria. However, there was little data available for this in the Tabuk region of Saudi Arabia. Moreover, almost all clinical bacteriology laboratories in Tabuk City do not carry out ESBL tests for gram-negative organisms. Therefore, this study will generate pilot results to study the prevalence of ESBL production Enterobacteriaceae in various clinical samples in Tabuk, the Kingdom of Saudi Arabia (KSA).

## Materials and methods

Study design

A cross-sectional study was conducted in the Clinical Microbiology laboratory of the Faculty of Medicine, University of Tabuk, Tabuk, KSA from March-May 2023. The isolates of Enterobacteriaceae (*E. coli, Klebsiella pneumoniae, Klebsiella oxytoca, Proteus mirabilis, Proteus vulgaris, Morganella morganii*, and *Citrobacter sp*) used for this study were collected from Microbiology laboratory of King Fahad Multispecialty Hospital, Tabuk, KSA. The isolates were collected as per the hospital policy. The bacteria belonging to the Enterobacteriaceae family were collected from the microbiology lab. The microbiology labs of the hospitals use standard methods for the identification of aerobic [11]. An institutional ethical clearance (approved no. UT-271-130-2023) was obtained from Local Research Ethics Committee (LREC) under the rules and regulations of the National Committee of Bioethics (NCBE), KSA.

Antimicrobial susceptibility testing

Aerobic antibiotic susceptibility testing was performed by using the Kirby-Bauer disk diffusion [12]. Briefly, 0.5McFarland turbidity inoculum was prepared with testing bacteria and spread over Muller-Hinton Agar (MHA) (HiMedia) and antimicrobial discs were applied to the plate. We have used cefotaxime (CTX: 30 μg), ceftazidime (CAZ: 30 μg), cefepime (FEP: 30 μg), cefoxitin (30 μg), gentamicin (GEN:10 μg), amikacin (30 μg) and amoxicillin (AMK: 30μg) in this study. The antibiotic used was from HiMedia-India.

Screening for potential ESBL-producing isolate

The bacteria were considered potential ESBL producers when they showed a ≤ 27 mm zone of inhibition with CTX (30 μg) and ≤22 mm with CAZ (30 μg) as recommended by Clinical and Laboratory Standards Institute (CLSI) guidelines [12]. *E. coli *ATCC 25922 (non-ESBL-producer), and *K. pneumoniae* 700603 (ESBL-producer) were used as control strains respectively.

Confirmation of ESBLs with combination disc test

The CAZ (30μg), CTX (30μg), FEP (30μg), and CAZ + clavulanic acid (30μg/10μg), CTX + clavulanic acid (30μg/10 μg), and FEP + clavulanic acid (30μg/10μg) were placed at appropriate distances on the Mueller-Hinton agar (MHA) plate, incubated as 37°C for 18 hours. The zone of inhibition difference of more than 5mm larger in clavulanic acid in comparison with a single ceftazidime or cefotaxime disc was considered as confirmed ESBL positive [13].

Data entry and analysis

We use Statistical Product and Service Solutions (SPSS) (IBM SPSS Statistics for Windows, Version 20.0, Armonk, NY) for data analysis. Data represented as number (percentage) (n (%)), otherwise indicated. The data were presented in tables and graphs.

## Results

Microbiological observations

A total of 90 aerobic bacteria were isolated from admitted patients of different wards (Table 1). The frequency of bacterial isolates from the different sources were shown in Table 1. *E. coli* was the most common isolate, accounting for 27.8%; followed by *Proteus mirabilis* and *Citrobacter sp* (15.6% each), *Klebsiella oxytoca* (12.2%), *Klebsiella pneumonia* (11.1%), *Proteus vulgaris* (10%) and *Morganella morganii* (7.8%). Among the sample, the majority of isolates were from urine (47.8%) followed by pus (25.6%) and the least from other body fluids (6.7%) (Table 2).

**Table 1:** Frequency of isolates N=90 Data were presented as n (%) unless otherwise indicated.

	N (%)
Escherichia coli	25 (27.8)
Klebsiella pneumoniae	10 (11.1)
Klebsiella oxytoca	11 (12.2)
Proteus mirabilis	14 (15.6)
Proteus vulgaris	9 (10.0)
Morganellas morganii	7 (7.8)
Citrobacter sp.	14 (15.6)

**Table 2:** Sample-wise distribution of isolates N=90 (total no of isolates, n=number in each category). Data were presented as n=number unless otherwise indicated. CSF: cerebrospinal fluid

	Urine	Blood	Pus	CSF	Body fluid
E. coli n=25	15	3	5	1	1
K. pneumoniae n=10	7	2	1	0	0
K. oxytoca n=11	6	1	1	1	1
P. mirabilis n=14	7	1	3	2	1
P. vulgaris n=9	3	2	3	0	1
M. morganii n=7	1	1	4	0	1
Citrobacter sp. n=14	4	2	6	1	1

Antibiotic resistance profile

**Figure 1:**
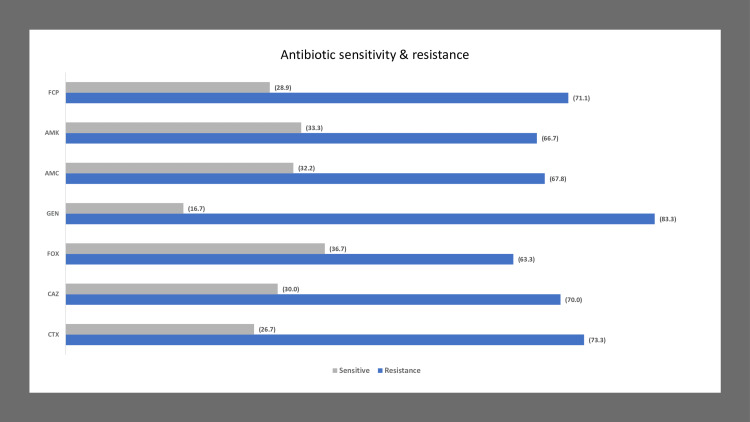
Average antibiogram of antibiotics CTX: cefotaxime, CAZ: ceftazidime, FOX: cefoxitin, GEN: gentamycin, AMC: amoxicillin, AMK: amikacin, FCP: cefepime. Data were presented as (%)=percentage unless otherwise indicated.

The antibiotic resistance of bacteria is presented in Figure 1.

On average the highest resistance was showed by GEN (83.3%) followed by CTX (73%), FCP (71.1%), CAZ (70%), AMC (67.7%), AMK (66.6%) and FOX (63.3%). The *E. coli *showed the highest average antibiotic resistance (73.7%) among all the antibiotics used followed by *P. mirabilis *(70.4%), *K. pneumoniae* (70%), *P. vulgaris* (69.8%), *M. morganii* and *Citrobacter* (69.4% both), and *K. oxytoca* (68.8%). The detailed antibiotic presentation of all isolated bacteria against the antibiotic were presented in Table 3.

**Table 3:** Antibiotic resistance profile of gram Enterobacteriaceae, n(%) CTX: cefotaxime, CAZ: ceftazidime, FOX: cefoxitin, GEN: gentamycin, AMC: amoxicillin, AMK: amikacin, FEP: cefepime. Data were presented as n (%) as number (percentage) and n=number unless otherwise indicated.

	CTX	CAZ	FOX	GEN	AMC	AMK	FEP
E. coli (n=25)	17 (68.0)	18 (72.0)	19 (76.0)	21 (84.0)	16 (64.0)	18 (72.0)	20 (80.0)
K. pneumoniae (n=10)	6 (60.0)	7 (70.0)	6 (60.0)	8 (80.0)	7 (70.0)	7 (70.0)	8 (80.0)
K. oxytoca (n=11)	8 (72.7)	6 (54.5)	7 (63.6)	9 (81.8)	7 (63.6)	8 (72.7)	8 (72.7)
P. mirabilis (n=14)	12 (85.7)	11 (78.6)	9 (64.3)	12 (85.7)	10 (71.4)	8 (57.1)	7 (50.0)
P. vulgaris (n=9)	6 (66.7)	6 (66.7)	5 (55.6)	7 (77.8)	7 (77.8)	6 (66.7)	7 (77.8)
M. morganii (n=7)	5 (71.4)	4 (57.1)	4 (57.1)	5 (71.4)	5 (71.4)	5 (71.4)	6 (85.7)
Citrobacter sp. (n=14)	12 (85.7)	11 (78.6)	7 (50.0)	13 (92.9)	9 (64.3)	8 (57.1)	8 (57.1)
Total=90	66	63	57	75	61	60	64

The magnitude of ESBL detection

Of all the isolates, 73.8% were positive in the screening of ESBL by disc diffusion method using CAZ zone of inhibition >22mm and CTX zone of inhibition >27mm. The phenotypic ESBL producers varied among isolated organisms. The lowest and highest intra-species positive for phenotypic ESBL were *K. oxytoca* (68.2%) and *P. mirabilis* (82.1) (Table 4).

**Table 4:** Magnitude of ESBL production: Screening result Data were presented as n (%) as number (percentage) and (%) as percentage unless otherwise indicated. ESBL: extended-spectrum beta-lactamase

ESBL study			
Screening	Ceftazidime	Cefotaxime	Average
E. coli	19 (76.0)	18 (72.0)	(74.0)
K. pneumoniae	7 (70.0)	7 (70.0)	(70.0)
K. oxytoca	8 (72.7)	7 (63.6)	(68.2)
P. mirabilis	12 (85.7)	11 (78.6)	(82.1)
P. vulgaris	7 (77.8)	6 (66.7)	(72.2)
M. morganii	5 (71.4)	5 (71.4)	(71.4)
Citrobacter sp.	12 (85.7)	10 (71.4)	(78.6)
	(77.1)	(70.5)	(73.8)

In the combination disk method (ESBL confirmatory), 45.9% were found positive by using CAZ/CAZ+CLV followed by FCP/FCP+CLV (43%) and CTX/CTX+CLV (41.1%) (Table 5).

**Table 5:** Magnitude of ESBL production: Confirmatory result Data were presented as n (%) as number (percentage) and (%) as percentage unless otherwise indicated. ESBL: extended-spectrum beta-lactamase

Confirmatory	E. coli	K. pleumoiniae	K. oxytoca	P. mirabilis	P. vulgaris	M. morganii	Citrobacter sp	Average
Ceftazıdıme/ceftazıdıme + clavulanıc acid	16 (64.0)	5 (50.0)	5 (45.5)	8 (57.1)	3 (33.3)	2 (28.6)	6 (42.9)	43.4%
Cefotaxime/cefotaxime + clavulanic acid	15 (60.0)	4 (40.0)	4 (36.4)	7 (50.0)	4 (44.4)	1 (14.3)	6 (42.9)	
Efepime/cefepime + clavulanic acid	15 (60.0)	5 (50.0)	4 (36.4)	8 (57.1)	3 (33.3)	2 (28.6)	5 (35.7)	
Average positivity among genus	(61.3)	(46.7)	(39.4)	(54.8)	(37.0)	(23.8)	(40.5)	

**Figure 2:**
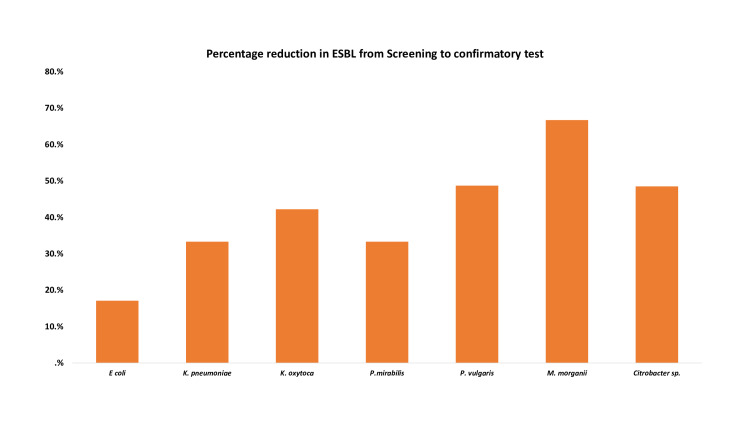
Percentage reduction of ESBL from screening to confirmatory ESBL: extended-spectrum beta-lactamase

Of all the isolates, the highest and lowest accounted for were *E. coli *61.3% and *M. morganii *(23.8%). There was a 41.2% average reduction in ESBL positivity from phenotypic to confirmatory test results. The highest reduction was observed among *M. morganii* (66.7%) and the least was observed in *E. coli* (17.1%) (Figure 2).

## Discussion

This study is a comprehensive analysis of the ESBL status of bacteria isolated from various clinical samples. Samples were predominantly from the urine followed by pus in our study. The group Enterobacteriaceae which produces ESBL was a very serious complication around the globe. The distribution of antibiotic broad-spectrum activity causes serious hindrances in the management of bacterial infections causing economic burden and sometimes life-threatening [14].

The present study observed that 57% of Enterobacteriaceae strains carried ESBL. The percentage of 7% showed a significantly greater size compared to the results found in past research carried out by Ethiopian scholars. The report provided information on the occurrence of ESBL-producing Enterobacteriaceae, indicating that the prevalence rates were at 34%. According to Siraj et al., there was a 4% increase in Jamma [15]. According to a study conducted by Mululem Ya et al. [16], 36% of individuals surveyed in Jamma exhibited a particular characteristic. The percentage rise in Harar was a staggering 33.3%, whereas in Adama it was 25% [17]. It is vital to adopt strong infection control measures due to the widespread occurrence of ESBL-producing Enterobacteriaceae in Addis Ababa. The present study reports 57.7% ESBL-producing Enterobacteriaceae, which was consistent with the reports of Bahir-Dar-Ethiopia [18]. Others also reported a very high prevalence of ESBL in Enterobacteriaceae members in various parts of the globe: Sudan [19], Cameroon [20], India [13] and Russia [21]. One of the key factors for the high prevalence of ESBL production is the widespread use of third-generation cephalosporins and insufficient antibiotic surveillance, inappropriate use of antibiotics, and inadequate implementation of infection control policy were the main reason for the high magnitude of ESBL. In contrast to the present investigation, the incidence rate of ESBL-producing Enterobacteriaceae in some European countries is comparatively low, this may be because the variance is due to the implementation of desperate measures in infection control within their countries and strict national policy. Moreover, it has been observed that our research result shows high compared to non-European nations such as Egypt (16%) [22]. The observed variation could plausibly be from various characteristics like the number of study subjects’ methodology. In our study, the prevalence of *K. pneumonia* (78.6%) and *E. coli *(52.2%) producing ESBLs which is consistent with the findings of Bahi Dar (*K. pneumonia* 69.8% and *E. coli* 55.2%) [18], Jimma (*K. pneumoniae *70.4%, *E. coli* 27.2%) [15], and in Uganda (*K. pneumonia *72.7% and *E. coli* 58.1%) [23].

In another study, the significant ESBL producers were *E. coli* compared with *K. pneumoniae* in Adama (*E. coli *51.5% and *K. pneumonia* 11.5%)[17], Burkina Faso (*E. coli *65.5% and *K. pneumonia* 26%) [24] and India (*E. coli* 61.4% and *K. pneumonia* 46.2%) [13]. The prevalence rate of Enterobacteriaceae produced by ESBL in pediatric populations below the age of 15 years was determined to be 74.1%, which is similar to the findings at TASH, Addis Ababa (78.5%) [25] and northeast of India (66.7%) [13], and also with rural Ghana (68%) [26].

The predominance of ESBL-producing Enterobacteriaceae was found in urine samples (47.8%), followed by pus samples (25.6%), blood samples (12.2%), and other sample types such as cerebrospinal fluid, and body fluid 10% and 6.7% respectively. Several studies reported blood samples were the most predominant origin of ESBL production in various geographical regions. In Bahra Dar, 72.7% of ESBL production was attributed to open wound pus [18]. On contrarily, blood samples were reported as the primary source of infection by ESBL in 75% of cases [24], and 87.8% in Iran [27]. This signifies that the infections by ESBL-producing strains of Enterobacteriaceae were a serious threat in the treatment of invasive bacterial infections. In various reports from different parts of the world, urinary specimens were identified as the primary source of ESBL-producing microorganisms from Central India (52.28% urine) [28], Uganda (64.9% urine, 474% urine) [23], and Bangladesh (70.4% urine, 16.5% blood) [29].

The study demonstrated high resistance to various drugs. This was in corresponding to the reports from Iran [27] and Nepal [30]. While comparing with the resistance pattern of antibiotics with the WHO published data, our resistance pattern is also justifying the reports of WHO on antibiotic resistance. This indicated the high resistance of drugs to commercially available and common drugs is becoming an alarming sign.

 Strength of the study

This is the preliminary report on the ESBL-producing Enterobacteriaceae from the city of Tabuk, Saudi Arabia. This research reveals the magnitude of ESBL dissemination and multidrug-resistant organisms (MDR) in Enterobacteriaceae and their resistance to other non-beta-lactam antibiotics.

Limitations

The study had some constraints in that carrying out the minimum inhibitory concentration (MIC) for all the ESBL-producing isolates obtained was considered laborious and time-consuming.

## Conclusions

The incidence of Enterobacteriaceae and MDR-isolated ESBL-producing organisms was high. *E. coli *and *K. pneumonia* were found to the frequent among ESBL-producing Enterobacteriaceae members with high resistance to several classes of antibiotics. Cefoxitin, amikacin and meropenem were the best available options for ESBL-positive Enterobacteriaceae. With the emergence of MDRs and ESBLs, the capacity of laboratory specialists to diagnose and monitor antibiotic resistance needs to be strengthened. We recommend the routine ESBL screening of bacteria and forming a strong hospital infection prevention policy.
